# A multi-arm multi-stage design for binary outcomes and application to tuberculosis

**DOI:** 10.1186/1745-6215-14-S1-O73

**Published:** 2013-11-29

**Authors:** Daniel Bratton, Patrick Phillips, Mahesh Parmar

**Affiliations:** 1 MRC Clinical Trials Unit Hub for Trials Methodology Research, London, UK

## 

Multi-arm multi-stage (MAMS) designs can greatly increase the speed and efficiency of treatment evaluation compared to separate trials of each new therapy. A class of MAMS designs with stopping guidelines only for lack-of-benefit have been developed for time-to-event outcomes and have been used to design several trials in oncology. In these designs, interim assessments can be made on an intermediate outcome which is on the causal pathway to the definitive primary outcome of the trial. Such designs can therefore incorporate both phase II and phase III in a single trial, further increasing the efficiency of treatment evaluation.

Tuberculosis (TB) is treated with combination regimens and, with many new or repurposed drugs currently in clinical development, better designs are needed to facilitate simultaneous testing of several new regimens in a single trial. To allow use of MAMS designs, we extend the methodology to binary outcomes, which are often used in TB trials.

We present methods for finding MAMS designs which have the desired pairwise type I error rate and power. Since there may be many such designs, choosing which to use can be tricky, as those which minimise the maximum or expected sample size are likely to perform poorly in practice. Designs which balance the two measures, known as admissible designs, may therefore be more appealing.

We give examples of admissible MAMS TB designs and demonstrate the savings in time and resources that could be made over conducting separate trials, particularly when using them to design seamless phase II/III trials.

